# Neurologic Effects of SARS-CoV-2 Transmitted among Dogs

**DOI:** 10.3201/eid2911.230804

**Published:** 2023-11

**Authors:** Dong-Hwi Kim, Da-Yoon Kim, Kyu-Sung Kim, Sang-Hoon Han, Hyeon-Jeong Go, Jae-Hyeong Kim, Kyu-Beom Lim, Dong-Hun Lee, Joong-Bok Lee, Seung-Yong Park, Chang-Seon Song, Sang-Won Lee, Yang-Kyu Choi, Yeun-Kyung Shin, Oh-Kyu Kwon, Do-Geun Kim, In-Soo Choi

**Affiliations:** Konkuk University, Seoul, South Korea (D.-H. Kim, D.-Y. Kim, S.-H. Han, H.-J. Go, J.-H. Kim, K.-B. Lim, D.-H. Lee, J.-B. Lee, S.-Y. Park, C.-S. Song, S.-W. Lee, Y.-K. Choi, I.-S. Choi);; Korea Brain Research Institute, Daegu, South Korea (K.-S. Kim, D.-G. Kim);; Daegu Gyeongbuk Institute of Science and Technology, Daegu (K.-S. Kim, D.-G. Kim);; Animal and Plant Quarantine Agency, Gimcheon, South Korea (Y.-K. Shin, O.-K. Kwon);; Konkuk University Zoonotic Diseases Research Center, Seoul (D.-H. Lee, J.-B. Lee, S.-Y. Park, C.-S. Song, S.-W. Lee, I.-S. Choi);; KU Center for Animal Blood Medical Science, Seoul (I.-S. Choi)

**Keywords:** COVID-19, 2019 novel coronavirus disease, coronavirus disease, severe acute respiratory syndrome coronavirus 2, SARS-CoV-2, viruses, respiratory infections, zoonoses, dogs, neurological model, neurodegenerative diseases, blood–brain barrier, infection transmission, South Korea

## Abstract

SARS-CoV-2 induces illness and death in humans by causing systemic infections. Evidence suggests that SARS-CoV-2 can induce brain pathology in humans and other hosts. In this study, we used a canine transmission model to examine histopathologic changes in the brains of dogs infected with SARS-CoV-2. We observed substantial brain pathology in SARS-CoV-2–infected dogs, particularly involving blood–brain barrier damage resembling small vessel disease, including changes in tight junction proteins, reduced laminin levels, and decreased pericyte coverage. Furthermore, we detected phosphorylated tau, a marker of neurodegenerative disease, indicating a potential link between SARS-CoV-2–associated small vessel disease and neurodegeneration. Our findings of degenerative changes in the dog brain during SARS-CoV-2 infection emphasize the potential for transmission to other hosts and induction of similar signs and symptoms. The dynamic brain changes in dogs highlight that even asymptomatic individuals infected with SARS-CoV-2 may develop neuropathologic changes in the brain.

Since SARS-CoV-2 was first reported in late 2019, infection has been observed primarily in humans; however, animals of various species have also been infected, partially because their angiotensin-converting enzyme 2 (ACE2) receptor is very similar to that of humans. Infected animals show clinical signs similar to those of humans, raising concerns about potential transmission of the virus between humans and animals (*1*,*2*). SARS-CoV-2 infection in dogs and cats affects the lungs and leads to pathologic changes (Figure 1). However, whether similar pathologic manifestations occur in the brain, as observed in humans, remains unclear.

**Figure 1:**
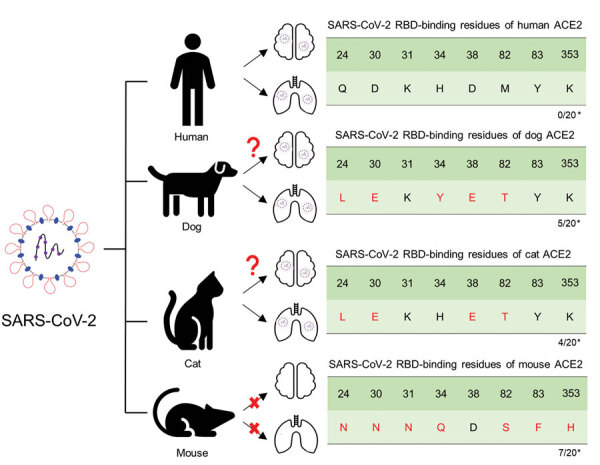
Schematic diagram representing susceptibility to SARS-CoV-2 infection in the lungs and brain of animals with potential for human transmission and homology of ACE2 amino acid sequences in study of the neurologic effects of SARS-CoV-2. *Number of mutations among the key 20 residues involved in interacting with the SARS-CoV-2 RBD. ACE2, angiotensin-converting enzyme 2; RBD, receptor-binding domain.

Close cohabitation of dogs and humans, and their high genetic similarity, has prompted investigations into dogs’ susceptibility to SARS-CoV-2 infection (*3*,*4*). Wild-type SARS-CoV-2 infection in dogs can induce formation of neutralizing antibodies, and low viral titers in dogs demonstrate seroconversion (*5*,*6*). Mutant strains of SARS-CoV-2 in dogs cause histopathologic changes in lung tissues and increased expression of muscle damage markers in the blood (*7*). ACE2 in dogs can bind to the receptor-binding domain of SARS-CoV-2, implying the possibility of cross-species transmission between humans and dogs (*8*). Genetic and epidemiologic studies have reported animal-to-human transmission of SARS-CoV-2 (*9*).

Reportedly, SARS-CoV-2 can cause neurologic signs and symptoms (e.g., headache, fatigue, and cognitive dysfunction) in human patients. Several cohort studies report strong correlations between SARS-CoV-2 and neurologic signs/symptoms (*10*–*13*). Furthermore, cortical thickness is reduced in SARS-CoV-2–infected patients, suggesting that SARS-CoV-2 can induce pathologic changes in the brain, which may be linked to the functional deficits noted in those patients. Considering the number of patients infected with SARS-CoV-2, the neurologic signs can lead to a potential wave of neurodegenerative diseases, which could pose an immense burden on society.

The etiology of SARS-CoV-2–induced neuropathologic changes is still elusive. However, clinical and experimental reports suggest that vascular damage and the resultant immune responses in the brain may be a major factor (*13*–*16*). Magnetic resonance imaging has detected white matter hyperintensities in SARS-CoV-2–infected patients, indicating damage to the blood–brain barrier (BBB) in this region and that potentially demyelinating pathologic changes can be induced (*13*). Other studies have revealed signs of neuroinflammatory responses, including activation of microglial cells and astrocytes (*14*,*15*). Moreover, damage to the brain vasculature and defects in the coagulation system have been demonstrated (*16*). The characteristic pathologies observed in human patients (e.g., vascular damage, demyelination, and neuroinflammatory responses) have also been observed in humanized mouse models.

We used a canine transmission model to investigate the susceptibility of dogs to SARS-CoV-2, specifically the Delta variant. The dogs were housed in a Biosafety Level 3 animal facility at Konkuk University Laboratory, Seoul, South Korea, where temperature, humidity, and light were carefully controlled. The study was approved by the Animal Research Center under the supervision of the Institutional Animal Care and Use Committee (accreditation no. KU22065) and the Institutional Biosafety Committee (accreditation no. KUIBC-2022-06) at Konkuk University. The absence of SARS-CoV-2 RNA and SARS-CoV-2 antibodies in dog serum was confirmed.

## Materials and Methods

Considering that SARS-CoV-2 infections cause neurologic effects in human and human ACE2 transgenic mice, and typically follow respiratory system infection, we used models mimicking the natural infection route. We intranasally infected dogs with the Delta variant, and virus subsequently was transmitted to contact dogs. We assessed detection of viruses in the brain and damage to the integrity of the BBB as well as activation of neuroimmune responses in the brain. To test whether SARS-CoV-2 can indeed induce neuropathologic changes in the brain, we also assessed further patterns of demyelination and axonal damage. We describe our methods here in brief; details are provided in the Appendix,

We purchased fifteen 6-month-old female conventional beagles from Orient Bio, South Korea (http://www.orient.co.kr) and classified them into 3 groups: control (n = 3), infection (n = 6), and contact (n = 6). The dogs in the infection and contact groups were housed in 6 cages, each measuring 800 mm wide × 900 mm deep × 800 mm high.

To mimic natural infection, we implemented 2 infection models: intranasally inoculated dogs and dogs infected via horizontal transmission. We anesthetized 6 dogs in the infection group with 0.3 mg/kg of alfaxalone and then intranasally inoculated each dog with 10^5^ PFU of SARS-CoV-2 Delta variant. After the dogs regained consciousness and acclimated to the environment, each infected dog was placed in a cage with a dog from the contact group. To control for any potential effects of the inoculation procedure or medium, we intranasally inoculated dogs in the control group with 500 µL of Dulbecco Modified Eagle Medium. Veterinarians visually examined the dogs for clinical signs, including neurologic signs.

With the infection model established, we next investigated the neuropathologic changes in the brain. First, we confirmed the existence of viral particles in the brain because it is logical to consider that viral particles can migrate to and replicate in the brain, which would directly damage the brain. To confirm the presence of viral nucleic acid we used quantitative reverse-transcription PCR, and to confirm the presence of viral particles we used immunofluorescence assays.

For use in additional experiments, at 4, 7, 11, 14, 18, 21, 25, 28, 32, and 35 days postinfection (dpi), we collected nasopharyngeal, oropharyngeal, and fecal swab and blood samples from all dogs while they were under sedation. At each timepoint in the early (10, 12, and 14 dpi) and late (38, 40, and 42 dpi) periods of infection, dogs were sedated and euthanized by intravenous injection of supersaturated KCl and performed necropsies (only 1 infected and 1 contact dog could be necropsied at each timepoint because of logistical constraints).

Samples underwent quantitative reverse transcription PCR, immunohistochemistry, immunofluorescence staining, ELISA, and plaque reduction neutralization test, as indicated (Appendix). We conducted all experiments in triplicate and express results as mean ±SD. We plotted dose-response curves, and we performed Student *t*-tests by using Prism 8.0.1 (Graphpad software, https://www.graphpad.com). We set statistical significance at p<0.05.

## Results

We detected no significant changes in objective measurements of the dogs (body weight and temperature). No dogs exhibited apparent neurologic signs or respiratory signs resembling COVID-19 (Appendix).

### Pathologic Changes in the Integrity of the BBB

**Figure 2:**
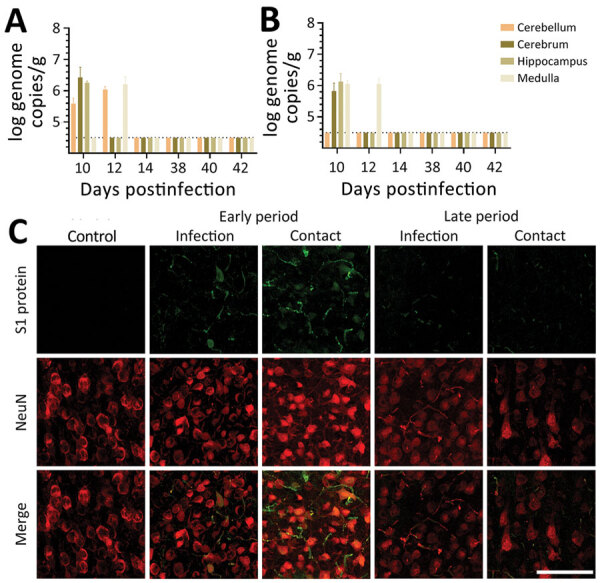
SARS-CoV-2 in the brain of dogs in study of the neurologic effects of SARS-CoV-2, showing transmission at an early stage of infection. A) Quantitative real-time PCR validation of the SARS-CoV-2 gene in the SARS-CoV-2–infected and contact groups of dogs, at each postinfection day. The dashed line indicates the regions where gene copy numbers of SARS-CoV-2 were considered negative. B) Representative fluorescent images of S1 protein (a marker of virus infection) and NeuN (a specific marker of neuronal cells) demonstrated SARS-CoV-2 infection in the canine brain sections of SARS-CoV-2–infected and contact dogs, at early and late days after infection. SARS-CoV-2–infected neuronal cells at the early stage of infection. At the late stage of infection, the presence of the virus appeared to diminish. Scale bars indicate 100 μm.

In this study, we detected viral RNA in the brain during the early infection period only, not during the late infection period (Figure 2, panel A). We confirmed colocalization of the viral particles with neuronal cells by using an immunofluorescence assay with an antibody specific to the spike protein of the virus. As for viral RNA, we also detected viral particles only during the early infection period (Figure 2, panel B). Our observations indicate that SARS-CoV-2 may infect the brain during the early infection period and may be cleared by the later infection period.

**Figure 3:**
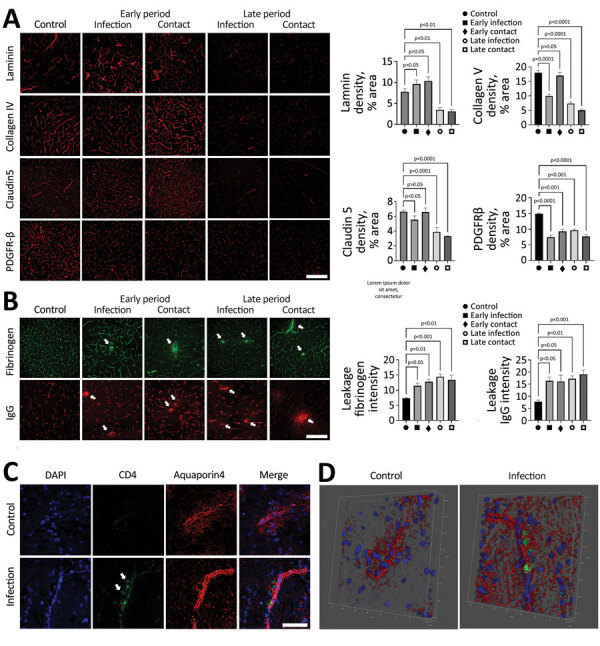
SARS-CoV-2 infection disrupting the blood–brain barrier (BBB) and immune cells infiltrating the brains of infected dogs in study of the neurologic effects of SARS-CoV-2 transmitted among dogs. A) Representative fluorescent images and statistical results of vascular markers including laminin, claudin 5, collagen IV, and PDGFR-β staining of canine brain sections derived from SARS-CoV-2–infected and contact dogs at early and late days after infection. Scale bar indicates 200 μm. B) Brain sections from SARS-CoV-2–infected and contact dogs stained with fibrinogen (green) and IgG (red), which represent BBB leakage staining, at early and late days after infection. Scale bar indicates 200 μm. (C) Representative fluorescent images of the brain sections stained with the CD4 (green) and aquaporin 4 (red), which are markers of CD4-positive T cells and astrocytic end foot, respectively. The CD4-positive cells were infiltrated in the brain parenchyma (white arrows) in the SARS-CoV-2–infected group. Scale bar indicates 50 μm. (D) Representative 3D images of CD4 (green) and aquaporin 4 (red). Statistical significance was determined using a 1-way analysis of variance. Data are presented as mean ±SEM.

Various reports suggest damaged brain vasculature in SARS-CoV-2–infected human patients, which is reported to be associated with the influx of peripheral molecules and activation of immune responses in the brain (*13*–*16*). In our study, we tested the pathologic changes in the canine brain vasculature by using an immunofluorescence assay with antibodies specific to the BBB compartments. For dogs infected with the virus, pathologic alterations in the BBB structure were noted, showing decreased signals of matrix proteins (laminin and collagen IV) and tight junction protein (claudin 5) (Figure 3, panel A). In dogs of both groups, those phenomena were prominently observed during late rather than early infection. In addition, PDGFR-β densities, which are markers for pericytes, were decreased in dogs of both groups during the early and late periods, indicating that the cellular components of the BBB were damaged by viral infection. Moreover, the infiltrations of fibrinogen and IgG were found in the parenchyma of the brain, indicating that viral infections breached the functional integrity of the BBB (Figure 3, panel B). Last, infiltration of CD4+ T cells was found in the brain by trespassing into the BBB matrix protein layer, suggesting severe damage to the BBB integrity and subsequent recruitment of these cells into the brain (Figure 3, panels C, D). Those observations indicate that SARS-CoV-2 infection could induce pathologic changes in the structural and functional integrity of the BBB. Such changes may allow entry of peripheral molecules and immune cells into the brain parenchyma during the early infection period. Collectively, the pathologic changes concur with the typical signs of small vessel disease (SVD).

### Neuroinflammatory Responses in the Brain

**Figure 4:**
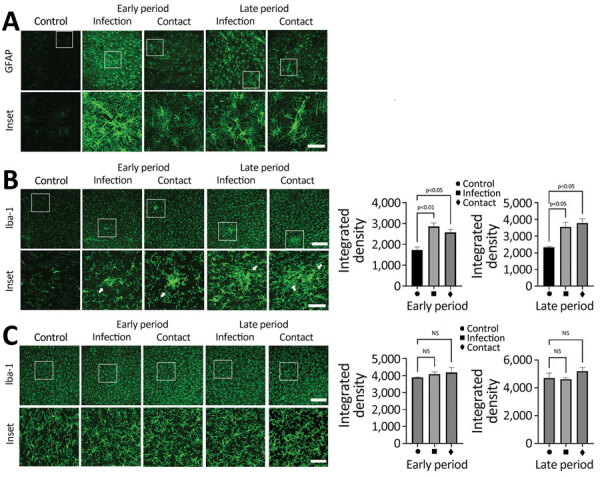
SARS-CoV-2 induces activation of microglial cells in the brain white matter in a region-specific manner in SARS-CoV-2–infected and contact dogs in study of the neurologic effects of SARS-CoV-2 transmitted among dogs. A) Representative fluorescent images of glial fibrillary acidic protein (activation astrocyte marker, green) staining of canine brain sections derived from SARS-CoV-2–infected and contact groups at early and late days after infection. Scale bars indicate 200 μm; in insets, 50 μm. B) Representative fluorescent images and statistical results of Iba-1 (a marker of microglia; green) staining of canine brain white matter sections derived from SARS-CoV-2–infected and contact dogs at early and late dpi. Scale bars indicate 200 μm; in insets, 50 μm. C) Representative fluorescent images and statistical results of Iba-1 (a marker of microglia, green) staining of canine brain gray matter sections derived from SARS-CoV-2–infected and contact dogs at early and late dpi. Scale bars indicate 200 μm; in insets, 50 μm. Statistical significance was determined using a 1-way analysis of variance. Data in graphs are presented as means ±SEM.

When we tested whether SARS-CoV-2–induced damage of the BBB can induce neuroinflammatory responses, we stained brain sections with markers for glial activation, including glial fibrillary acidic protein and Iba-1, which are markers for activated astrocytes and microglial cells. Glial fibrillary acidic protein showed a statistically significant increase in the brain white matter of dogs in the infection and contact groups at the early and late periods, suggesting potential proinflammatory conditions in the brain (Figure 4, panel A). When we tested activation of microglial cells (another major component of innate immune responses in the brain) by staining brain sections with an antibody specific to Iba-1 (a marker of activated microglial cells), we observed a significant increase in Iba-1–positive signals in the brain white matter of dogs from both groups during the early and late periods (Figure 4, panel B). However, we did not observe such increases in the gray matter, suggesting that the microglial cell activations were specific for white matter (Figure 4, panel C). Of note, we observed activated microglial cell clustering in several spots in the white matter from dogs in both groups. Overall, those observations suggest that BBB disruption mediated by infection with SARS-CoV-2 could elicit neuroinflammatory responses and further contribute to the progression of neurodegenerative pathology in canine brains.

### Typical Signs of SVD-mediated Axonopathy

**Figure 5:**
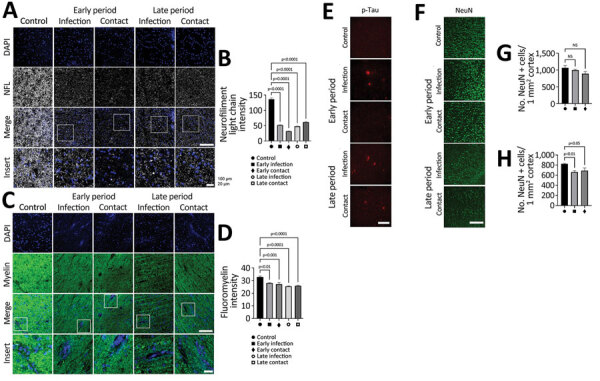
SARS-CoV-2 infection causing perivascular demyelination in the brain in dogs in study of the neurologic effects of SARS-CoV-2 transmitted among dogs. A, B) Representative fluorescent images and statistical analysis results of NFL (a marker of neurofilament light chain; gray) staining of canine brain white matter sections derived from SARS-CoV-2 infected and contact dogs at early and late dpi. Scale bar indicates 200 μm. Overall images from infected dogs demonstrate irregular axonal morphology compared with that of control dogs. Single-layer slice images. Scale bar indicates 20 μm. C, D) Representative fluorescent images and statistical analysis results of myelin (fluomyelin; green) and DAPI (blue) staining of canine brain white matter sections derived from SARS-CoV-2–infected and contact dogs at early and late dpi. Scale bar indicates 200 μm. E) Representative fluorescent images of p-tau (red) staining of canine brain gray matter sections derived from SARS-CoV-2–infected and contact dogs at early and late dpi. Scale bar indicates 200 μm. F–H) Representative fluorescent images (F) and statistical analysis results (G, H) of NeuN (a marker of neuron; green) staining of canine brain gray matter sections derived from SARS-CoV-2–infected and contact dogs at early (G) and late (H) infection. Scale bar indicates 200 μm. Statistical significance was determined using a 1-way analysis of variance. Data are presented as mean ±SEM. NS, not significant.

The early signs of SVD-mediated brain neurodegeneration are pathologic changes in axons and demyelination. To verify whether SARS-CoV-2 can induce these pathologic changes, we stained brain sections with antibodies against the neurofilament light chain (NFL). The intensities of NFL staining were significantly lower in the brain white matter of dogs in both groups at the early and late periods than in the uninfected control dogs (Figure 5, panels A, B). In addition, the pathologic changes in the structure and integrity of the NFL were evidenced by swelling and irregularity**.** Decreased NFL intensities were more severe in perivascular regions, as typical signs of SVD-mediated axonopathy. 

Because demyelination is another hallmark of SVD, we assessed demyelination by using fluomyelin, a fluorescent marker for myelin. We observed a significantly lower intensity of fluomyelin in the brain white matter of dogs in the infection and contact groups during the early and late periods; NFL patterns were similar in both groups (Figure 5, panels C, D). Those changes were more evident in the perivascular area, identical to the NFL lesions**.** The axonopathy-like changes observed at the perivascular area of the white matter could be a consequence of SVD induction.

### Pathologic Signs of Neurodegenerative Diseases, Represented by Tauopathy

To assess production of Aβ aggregation and test whether SVD-induced neuropathologic changes can further cause neurodegenerative signs, we stained brains with an amyloid β (6E10) antibody. However, we did not observe formation of Aβ aggregates in the brains of any dogs during the early or late periods (Appendix Figure 10, panel A). Next, to assess tauopathy in the virus-infected brains, we stained brain sections with different types of phosphorylated tau. Using a p-Tau 181 antibody, we did not observe positive signals for phosphorylated tau in any dogs (Appendix Figure 10, panel B). However, we detected phosphorylation of tau at Ser202/Thr205 by using an AT8 antibody in the brains of dogs in the infection group during the early period and dogs in the infection and contact groups during the late period. Those results suggest that SARS-CoV-2 infection could induce accumulation of the pathologic form of tau in a site-specific manner (Figure 5, panel E). Last, we used the number of neuronal cells to determine whether those pathologic neurodegenerative signatures are associated with loss of neuronal cells. We did not observe statistically significant changes in the number of cortical neurons in the brains of dogs from the infection and contact groups during the early period (Figure 5, panels F–H). However, we observed decreased numbers of neuronal cells in the infection and contact groups during the late period**.** Therefore, degenerative changes such as tauopathy and decreased numbers of neuronal cells in the virus-infected brains seemed to be induced after elicitation of pathologic drivers, including BBB damage, glial activation, and axonopathy, as consequences of SVD.

## Discussion

Overall, our study demonstrates solid experimental evidence that SARS-CoV-2 can infect dogs and be transmitted to others by direct contact, producing pathologic brain changes even without prominent signs. Pathologic changes in the lung and brain were observed in dogs of both groups, providing additional evidence of virus transmission. Of note, SARS-CoV-2 infection has been reported to cause long-term pathologic effects even after the virus is cleared from the main organs of the body (*17*). Our study provides evidence that SARS-CoV-2 infection can damage the brain as well as the lungs in dogs at early and later stages of infection, suggesting a high potential for a long-lasting COVID-19–like syndrome to develop in affected dogs.

We detected SARS-CoV-2 in secretions from the nasopharynx and oropharynx of dogs in both the infection and contact groups, albeit at a low percentage. Remarkably, we found that the viral titers were higher in the nasal and oral mucosa of dogs in the contact group than in those in the infection group. That finding could be attributed to the role of the nasal and oral cavities as routes of virus entry for the contact group, resulting in higher replication of the virus at these entry points (*18*). We observed that during the early stages of infection, dogs in the contact group exhibited more severe inflammatory responses in the trachea and bronchioles than did those in the infection group. Those findings are consistent with results of previous studies that have shown that contact transmission can result in higher levels of virus titers and lead to more rapid onset of pathologic changes in the upper respiratory tract (*19*,*20*).

Seroconversion in dogs after SARS-CoV-2 infection was observed as early as 4 dpi; the rapid seroconversion may be associated with the absence of clinical signs (*21*). Antibody levels peaked a few days later in dogs in the contact group than in dogs in the infection group, suggesting later virus transmission from the virus-infected dogs. Neutralizing antibody titers against SARS-CoV-2 strongly correlate with antibody titers of the spike protein, highlighting the spike protein as a crucial target for the humoral immune response (R value >0.7; p< 0.001).

The lung alveolar septum of infected dogs was thickened overall, caused by infiltration of immune cells that indicate interstitial pneumonia (e.g., mononuclear cells, neutrophils, and macrophages) (*22*,*23*), associated with the presence of neutrophil elastase–positive cells and Iba-1–positive cells. Neutrophil elastase and Iba-1 levels increase in response to SARS-CoV-2 infection (*22*,*24*). Neutrophil elastase–positive and Iba-1–positive cells were found infiltrated around the blood vessels, indicating perivasculitis in SARS-CoV-2 infected dogs as in other hosts (*22*,*25*).

Brain damage induced by viral infection has been reported from various nonneurotrophic viruses, including HIV (*17*,*26*). Contrary to previous beliefs, accumulating evidence argues that SARS-CoV-2 can induce pathologic changes in the brains of several hosts, including humans, although the detailed mechanisms of those pathologies are still elusive. We analyzed the histopathologic changes in the brains on the basis of those uncertain arguments. Of note, we observed drastic pathologic changes in the dog brains, although the animals did not exhibit any neurologic signs**.** One of the most prominent features of brain pathology was the BBB damage observed during early infection and maintained until later infection. Typical features of vascular damage observed from SVD were changes in the level of tight junction proteins, decreased levels of laminin, and reduced pericyte coverage (*27*). SVD involves functional and structural dysfunctions of the brain vasculature, demonstrating white matter hyperintensities, microbleeding, and increased perivascular spaces. Moreover, SVD induces increased influx of peripheral blood factors and immune cells into the brain. Our finding of initial pathologic features of the BBB commonly observed in SVD in the brains of SARS-CoV-2–infected dogs strongly supports our hypothesis that the virus can induce SVD in dog brains. Several human studies supporting our observations also reported these pathologic features of the BBB similarly found in SVD (*26*,*28*,*29*).

SVD induces an influx of peripheral molecules and a favorable environment for producing large amounts of reactive oxygen species that activate microglial cells and astrocytes—hallmarks of neuroinflammatory responses. In our study, we specifically observed activation of glial cells in the white matter of the brains of the SARS-CoV-2–infected dogs, suggesting the neuroinflammatory conditions that SARS-CoV-2–mediated SVD might induce. The activation of microglial cells has also been observed in humanized ACE2 mice and brains from different animal species, including nonhuman primates (*30*,*31*). The marked axonopathy in the white matter and the preferentially increased activity of glial cells in this region strongly suggest correlations between the glial activation and development of axonopathy potentially mediated by development of SVD by SARS-CoV-2 infection. Furthermore, activation of the astrocytes and microglial cells was maintained up to 40 dpi, even when the virus was cleared from the brain. That finding strongly suggests that the glial cells activated by SARS-CoV-2 potentially harm axons or other components of neuronal cells, even when virus is absent in the brain. That topic could be the focus of future research that requires further in vitro/in vivo studies to reveal the mechanistic link between glial activation and neuronal damage mediated by SARS-CoV-2 infection.

Tau phosphorylation is the hallmark of Alzheimer’s disease. Tau is the family of the microtubule-associated protein tau and functions in the delivery of synaptic vesicles required for synaptic transmission; phosphorylation of tau causes loss of this property, but the mechanism remains elusive (*32*). There are multiple phosphorylation sites on the tau protein, and our study shows the specific phosphorylation of Ser202/Thr205, detected by using the AT8 antibody (*33*). Detection of phosphorylated tau suggests a high probability of developing signs of neurodegenerative diseases in the SARS-CoV-2–infected brain. A recent study has shown the correlation between the development of SVD and the accumulation of phosphorylated tau, supporting the finding that development of phosphorylated tau could be oriented by SARS-CoV-2–associated SVD (*34*,*35*).

Long-term brain damage induced by SARS-CoV-2 has become a major topic for research of long COVID syndromes in humans (*36*). It has been reported that ≈10% of SARS-CoV-2–infected persons experience neurologic signs/symptoms, suggesting potential neurotrophic characteristics of this virus (*37*). According to recent retrospective studies that used UK Biobank data (https://www.ukbiobank.ac.uk), shrinkage in the brain cortex and decreased cognitive function have been reported for human patients after recovery from SARS-CoV-2 infection (*38*). Moreover, postmortem human brain tissue analysis demonstrated increased activity of glial cells, proinflammatory immune responses, neuronal damage, and BBB damage, enabling peripheral immune cells to infiltrate, strongly suggesting neuropathologic changes induced by SARS-CoV-2 infection (*39*). However, those pathologic changes were analyzed mainly in brain samples from patients with severe neurologic sign/symptoms; neuropathologic changes in asymptomatic patients are still elusive. From that perspective, our study has value as translational research to predict neuropathologic changes in the early phase of asymptomatic SARS-CoV-2 infection in humans because we have observed the kinetic pathologic changes in the brains of dogs that did not show any neurologic signs. Compared with other animal models, dogs are genetically similar to humans and their brain structures are similar to those of humans, making our extrapolation more reliable. According to our results, the brains of dogs infected with SARS-CoV-2 demonstrate severe BBB disruptions and consequent SVD-like pathologic signs, including axonopathy, glial activation, and potential neurodegenerative changes even without neurologic signs. That evidence strongly suggests that even asymptomatic SARS-CoV-2 patients might have neuropathologic changes in their brains, which could develop into severe neurologic disorders later in life.

Among the merits of our study in terms of translational research of SARS-CoV-2–induced neuropathologic changes, we compared 2 infection routes: direct intranasal infection and horizontal transmission models that can mimic more natural infection routes. With that comparison, we determined that neuropathologic changes can be induced via both exposure routes, providing valuable information that owners of companion animals potentially face SARS-CoV-2–associated neurologic disorders. Second, we studied dogs, which are a more advanced species than rodents, to provide neuropathologic data that are closer to data for humans and more relevant. Moreover, our data suggest that neuropathologic changes can be induced in dogs. Last, we found that the neuroinflammatory responses were more prominently observed in the white matter area than the gray matter area, suggesting that the neuroinflammatory responses induced by SARS-CoV-2 differ by brain region. Overall, these data can be used as translational research data to interpret the potential neuropathologic changes that may be observed in humans.

Appendix Additional methods and results from study of neurologic effects of SARS-CoV-2 transmitted among dogs.
