# Supplementary material for: Neurologic Effects of SARS-CoV-2 Transmitted among Dogs
Source: Emerg Infect Dis. 2023 Nov;29(11):2275–84. doi: 10.3201/eid2911.230804 (PMC10617347; doi:10.3201/eid2911.230804)
Supplement: Appendix — Additional methods and results from study of neurologic effects of SARS-CoV-2 transmitted among dogs. [file 23-0804-Techapp-s1.pdf]

Article DOI: https://doi.org/10.3201/eid2911.230804

EID cannot ensure accessibility for supplementary materials supplied by authors. Readers who have difficulty accessing supplementary content should contact the authors for assistance.

Neurologic Effects of SARS-CoV-2 Transmitted among Dogs

Appendix

MATERIALS AND METHODS

Virus amplification, experimental design, and sample collection

SARS-CoV-2 delta variant (GK clade, AY.69 lineage, NCCP No. 43409) was obtained from the National Culture Collection for Pathogens in South Korea. The virus was amplified on Vero76 cells and titrated using plaque forming assay. The dogs were classified into three groups: MOCK (n = 3), infection (n = 6), and contact (n = 6). The dogs in the infection and contact groups were housed in six cages, with each cage measuring 800W × 900D × 800H mm. One dog from the infection group and one dog from the contact group were kept together in each cage. Six dogs in the infection group were anesthetized with 0.3 mg/kg of alfaxalone (Alfaxan® Multidose, Jurox, Australia) and then inoculated with 10^5 PFU of SARS-CoV-2 delta variant through the nasal cavity. After regaining consciousness and acclimating to the environment, each infected dog was placed one-on-one in a cage with a dog from the contact group. The MOCK control group was inoculated with 500 µL of Dulbecco's Modified Eagle Medium into the nasal cavity to control for any potential effects of the inoculation procedure or medium. The clinical signs, including neurological signs in the dogs, were visually evaluated by veterinarians. At 4-, 7-, 11-, 14-, 18-, 21-, 25-, 28-, 32-, and 35-days post-infection (dpi), nasopharyngeal, oropharyngeal, and fecal swabs, as well as blood samples, were collected from all dogs under sedation for further experiments. At the early (10, 12, and 14 dpi) and late (38, 40, and 42 dpi) periods of infection, the dogs were euthanized by intravenous injection of supersaturated KCl under sedation, and autopsies were performed. Due to logistical constraints, only one infected and one contact dog was autopsied daily.

Statistical analysis

All experiments were conducted in triplicate and are expressed as the mean ± SD. Dose-response curves were plotted, and Student's t-tests were performed using PRISM 8.0.1 (Graphpad Software, CA); statistical significance was set at $p < 0.05$.

Chemicals and Reagents

The following antibodies were used: anti-fibrinogen (1:500, Dako, A0080), anti-Dog IgG (IFA; 1:500, Invitrogen, SA5-10309), anti-Dog IgG (ELISA; 1:1000, GeneTex, GTX77013), Dog IgM (1:1000, GeneTex, GTX77081), anti-Dog IgA (1:1000, GeneTex, GTX77075), HRP-conjugated anti-Goat IgG (1/5000, Abcam, ab97110), anti-NeuN (1:500, CST, 12943s), anti-SARS-CoV-2 spike protein (1:500, Invitrogen, MA5-36247), anti-GFAP (1:500, CST, 3670s), anti-neutrophil elastase (1:200, Abcam, ab68672), anti-Iba-1 (1:500, FUJIFILM, 019-19741), anti-FluoroMyelin (1:500, Invitrogen, F34652), anti-NFL (1:500, CST, 2837s), anti-phospho Tau (1:500, Thermo, MN1020), anti-6E10 (1:500, Bio-legend, 803001), anti-CD31 (1:100, MyBiosource, MBS249056), anti-AQP4 (1:500, CST, 59678), anti-Laminin (1:500, Abcam, ab11575), anti-Claudin5 (1:500, Invitrogen, 35-2500), anti-Collagen IV (1:500, Abcam, ab6586), anti-PDGFRβ (1:500, CST, 3169s), and CD4 (1:100 dilution, R&D system, MAB2410). The following recombinant antigens were used: recombinant SARS-CoV-2 spike S1 protein (GeneTex, GTX135817-pro) and recombinant SARS-CoV-2 nucleocapsid protein (GeneTex, GTX135357-pro).

Reverse transcription-quantitative polymerase chain reaction (RT–qPCR)

Nasopharyngeal, oropharyngeal, and fecal swabs were resuspended in 150 μL of phosphate-buffered saline (PBS). Blood samples were collected using BD Vacutainer® SST™II Advance (BD, UK) and centrifuged at 3,000 × *g* to obtain serum samples. Viral RNA was extracted from the nasopharyngeal, oropharyngeal, and fecal swabs, and serum using the RNeasy Mini RNA isolation kit (Qiagen, Germany), following the manufacturer's instructions. RNA from canine tissue was extracted using the TRIzol reagent (Invitrogen, 15596018). Chloroform was added to the mixture and then centrifuged at 12,000 × *g* for 15 min. The supernatant was combined with isopropanol at a ratio of 1:1 and incubated at room temperature for 10 min. The mixture was centrifuged at 12,000 × *g* for 10 min, and the supernatant was discarded. The RNA pellet was washed twice with 75% ethanol in nuclease-free water. The ethanol was removed, and the pellet was air-dried for 10 min. The RNA pellet was resuspended in 30 μL of nuclease-free

water and reverse transcribed using the High-Capacity cDNA Reverse Transcription kit with RNase inhibitor (ABI, 4374966). Real-time RT-qPCR was conducted using the KAPA SYBR FAST kit (Roche, KK4602). The PCR mixture contained KAPA SYBR FAST qPCR Master Mix (2X) Universal, gene-specific primers for the SARS-CoV-2 M gene, and 50X ROX High. Thereafter, 20 ng of cDNA product was diluted in the PCR mixture. Amplification was performed using the QuantStudio 5 machine. Thermal cycling conditions consisted of pre-incubation at 95°C for 3 min, followed by 40 cycles of amplification at 95°C for 10 s. The melting temperature of the PCR product was determined via melting curve analysis, which was performed by heating the PCR product from 65°C to 97°C and then cooling it at 40°C for 10 s. The viral GE copy numbers were calculated from a standard curve generated with plasmid DNA containing the target sequence of the M gene. The relative fold changes in control, infection, and contact groups were calculated using the comparative threshold cycle method. The following gene sequences were used in this study: SARS-CoV-2 M gene: Forward primer, 5′-GGYTCTAARTCACCCATTCA -3′; Reverse primer, 5′-TGATACTCTARAAAGTCTTCATA -3′

Immunohistochemistry (IHC)

IHC was performed for Iba-1 and neutrophil elastase. Formalin-fixed, paraffin-embedded tissues were processed into 5 μm sections and mounted on silane-coated slides. The sections were then deparaffinized in xylene, rehydrated in graded ethanol, and washed with distilled water (DW). Antigen retrieval was achieved by pressurizing the sections in Na-citrate buffer (pH 6.0) for 10 min, followed by cooling in iced water and washing thrice with PBS. Thereafter, the sections were incubated with 3% hydrogen peroxide prepared in methanol for 10 min at room temperature to block endogenous hydrogen peroxidase activity. After washing thrice with PBS, the sections were treated with normal goat serum (Vectastain Elite ABC-HRP Kit, Peroxidase (Rabbit IgG); Vector Laboratories, US) for 30 min to block non-specific binding of antibodies. The sections were then treated with the primary antibodies and incubated overnight at 4°C (see Table 1 for details). The sections were washed thrice with PBS, incubated with biotinylated goat anti-rabbit IgG (Vectastain Elite ABC-HRP Kit, Peroxidase (Rabbit IgG); Vector Laboratories, US) for 1 h at room temperature, rewashed thrice with PBS, and incubated with ABC reagent (Vectastain Elite ABC-HRP Kit, Peroxidase (Rabbit IgG); Vector Laboratories, US) for 30 min at room temperature. The primary antibody, biotinylated secondary antibody, and avidin-biotin

horseradish peroxidase complex were visualized using the DAB/Ni peroxidase substrate kit (Vector Laboratories, US). Finally, the sections were counterstained with Gill's Hematoxylin II, dehydrated in graded alcohol series, and mounted.

Immunofluorescence staining

The brains were fixed in 4% paraformaldehyde (PFA) overnight and incubated in 30% sucrose for 48 h before embedding and freezing in an optimal cutting-temperature compound (Tissue-Tek). Brain sections were cut into 20 μm sections and stored at −20°C. Before immunostaining, the slides were permeabilized with 1% Triton X-100 in PBS for 20 min and blocked with 5% normal goat serum in PBS for 1 h. For immunostaining of the brain vascular and peripheral immune cells, the canine brain tissues were sectioned to 50 μm, maintained in 0.5% NaH_2PO_4 until use, and stored at 4°C. Antigen retrieval was performed by incubating the sections in citrate buffer, pH 6.0, at 80°C for 30 min. Primary antibodies were diluted in 1% Triton X-100 in PBS containing 5% normal goat serum and incubated overnight at room temperature. The following day, the slides were washed five times in dPBSm, incubated with fluorescently conjugated secondary antibodies for 1 h at room temperature, and diluted 1:500 fold in 5% normal goat serum in PBS. After washing with dPBS, the slides were counterstained with DAPI and coverslipped with Fluoromount-G (Electron Microscopy Science). Images were acquired with a Leica Confocal microscope (SP8) and reconstructed using LAS X software with maximal projection intensity (MPI).

Enzyme-linked immunosorbent assay (ELISA)

Antibody titers specific for the SARS-CoV-2 S protein and N protein were determined using ELISA. Serum samples were diluted 1000-fold for IgG and 10000-fold for IgM and IgA and then transferred to microplate wells coated with recombinant SARS-CoV-2 S protein or N protein at a concentration of 0.2 μg/mL. After washing five times with PBS, the plate was incubated with anti-IgG, anti-IgM, or anti-IgA antibodies for 1 h and 30 min. After washing five times with PBS, the plate was incubated with 5000-fold-diluted HRP-conjugated anti-goat IgG for 1 h. After washing five times with PBS, the colorimetric reaction was initiated by adding tetramethylbenzidine (TMB) and terminated by adding 2M H2SO4. Optical density (OD) was measured at 450 nm using an ELISA reader.

Plaque reduction neutralization test

Neutralizing antibodies were detected by performing a plaque reduction neutralization test. In this assay, serum was diluted 10-fold, and 50 μL of the diluted serum was mixed with 30 PFU/50 μL of the SARS-CoV-2 delta variant. The mixture was then incubated at 37°C for 1 h and transferred to a 6-well plate containing cells at 90% confluency. The cells were infected with the virus mixture for 1 h and washed thrice with PBS. The cells were overlaid with 2% DMEM and 0.6% agarose. After four days of infection, the number of plaques caused by viral infection was counted, and the plaques formed in the presence of serum from the negative control group were compared. The presence of neutralizing antibodies was confirmed if the percentage of plaques formed in the presence of serum from the experimental group was lower than that of the negative control group. Data are presented as mean ± SD.

Establishment and verification of canine SARS-CoV-2 infection and transmission model

SARS-CoV-2 RNA was detected in nasopharyngeal and oropharyngeal swabs of both groups from 4–14 days post-infection (dpi). All dogs in both groups had detectable SARS-CoV-2 RNA in their serum and feces at 14 dpi, and in some dogs, the virus persisted until 18 dpi (Appendix Figure 1). Clinical signs such as weight loss, increased body temperature, and cough were carefully monitored during the experimental period. However, all dogs showed no clinical signs of COVID-19 (Appendix Figure 2). To confirm the distribution of SARS-CoV-2 in the lungs, lung tissue samples were collected through autopsy from an infected dog and a contact dog on a determined day at two different periods set as an early period (10, 12, and 14 dpi) and a late period (38, 40, and 42 dpi) of infection. Six lung lobes were collected from each dog. SARS-CoV-2 RNA was detected in two dogs during the early infection period: one in the infected dog (10 dpi, right accessory lobe) and one in the contact dog (12 dpi, right cranial and left cranial lobes). Immunohistochemical analysis of the viral-positive tissues revealed the presence of viral antigens (Appendix Figure 3).

The histopathological changes were confirmed in the lung tissues obtained from dogs in both infection and contact groups. The alveolar septa of the lung tissues in all dogs were thickened by interstitial infiltration of inflammatory cells (neutrophil elastase$^+$ or Iba-1$^+$ cells)

during the early infection period, which persisted to the late period (Appendix Figure 4, panel A, and Appendix Tables 1 and 2). The percentage of parenchymal tissue in the lungs was calculated by the methods described in Appendix Figure 5. The analysis was performed on randomly selected zones within the six lobes of the lungs obtained from all individuals. All experimental groups exhibited a significant increase in lung parenchymal areas compared with the negative control group. The parenchymal areas in the infection group dogs were significantly wider during the late infection period than in the early period. The parenchymal areas of the contact group were larger in the early infection period than in the late period (Appendix Figure 4, panel B, and Appendix Table 3). Lesions exhibiting thickened alveolar septa were confirmed to have infiltration of neutrophils and other mononuclear cells, considered a diagnostic marker of interstitial pneumonia (Appendix Figure 4, panel C). Distinct histopathological changes were evident in the dogs of the contact group, including bronchus-associated lymphoid tissue (BALT), which may have been formed through antigen priming in the early infection period. Furthermore, inflammatory cell infiltration was observed in the trachea and bronchiole tissues. In the late period, there was evidence of infiltration by neutrophil elastase-positive cells or Iba-1-positive cells in those tissues of the contact group dogs (Appendix Figure 6). The infiltrated inflammatory cells were observed around the pulmonary blood vessels in the infection and contact groups. This phenomenon was observed during both the early and late infection periods. Immunostaining analysis confirmed the involvement of neutrophil elastase-positive cells and Iba-1-positive cells in the perivascular inflammatory response (Appendix Figure 7).

Serological evidence of humoral immune response to SARS-CoV-2 infection in dogs

To further investigate whether there were immunological changes in response to viral infection, we examined the formation of humoral immunity against SARS-CoV-2. For this, antibody tests for anti-SARS-CoV-2 IgM, IgG, and IgA were conducted using ELISA after coating SARS-CoV-2 spike (S) and nucleocapsid (N) proteins to determine seroconversion to SARS-CoV-2 infection in both groups. In the infected group, anti-S IgM levels peaked at 11 dpi and decreased afterward, whereas the peak and subsequent decrease were approximately 14 dpi in the contact group. Anti-S IgG was also detected from 4 dpi, and its levels remained high until 35 dpi, unlike anti-S IgM, which declined after the early period. The blood anti-S IgA of the

experimental group showed higher antibody values than that of the MOCK group. However, anti-S IgA exhibited relatively low OD values compared with anti-S IgM and IgG (Appendix Figure 8, panel A). Anti-N antibodies showed a similar trend to that of anti-S antibodies but with a higher background signal (Appendix Figure 8, panel B). These results demonstrate that seroconversions effectively occurred in dogs from both groups—intranasally infected with the SARS-CoV-2 delta variant and contacted from the infected dogs. The neutralizing activities of the serum antibodies against the SARS-CoV-2 delta variant were determined with 10-fold serially diluted serum samples. A decrease in plaque formation was observed in all infected and contact dogs, indicating that the antibodies present in the serum possess neutralizing ability against the virus (Appendix Figure 8, panel C). The plaque reduction rates of the infection group were over 60% in all dogs except one at 7 dpi, while the highest reduction rates observed in the contact group were only 32.63% on average. By 28 dpi, all dogs in both groups showed more than 90% plaque inhibition (Appendix Table 4). When a correlation between the plaque reduction ability and the anti-S Ig or anti-N IgG was analyzed, the reduction of plaque formation was significantly associated with anti-S IgG but not anti-N IgG titers (Appendix Figure 9). By conducting these verification procedures, we established that this infection model is suitable for analyzing neuropathological changes in the canine brain.

Appendix Table 1. Comparison of Iba-1 positive ratios between five study groups: results of t-tests

**Group** (Mean ± SD)	**MOCK** (1.599 ± 1.015)	**Infection, Early period** (4.248 ± 2.626)	**Contact, Early period** (3.272 ± 0.983)	**Infection, Late period** (4.950 ± 3.092)	**Contact, Late period** (4.227 ± 3.527)
**MOCK** (1.599 ± 1.015)	-	** $p = 0.0025$	*** $p < 0.001$	** $p = 0.0011$	* $p = 0.0185$
**Infection, Early period** (4.248 ± 2.626)	** $p = 0.0025$	-	$p = 0.1491$	$p = 0.4682$	$p = 0.9842$
**Contact, Early period** (3.272 ± 0.983)	*** $p < 0.001$	$p = 0.1491$	-	* $p = 0.0352$	$p = 0.2762$
**Infection, Late period** (4.950 ± 3.092)	** $p = 0.0011$	$p = 0.4682$	* $p = 0.0352$	-	$p = 0.5180$
**Contact, Late period** (4.227 ± 3.527)	* $p = 0.0185$	$p = 0.9842$	$p = 0.2762$	$p = 0.5180$	-

*$p < 0.05$, **$p < 0.01$, and ***$p < 0.001$

Appendix Table 2. Comparison of neutrophile elastase positive ratios between five study groups: results of t-tests

**Group** (Mean ± SD)	**MOCK** (3.285 ± 1.905)	**Infection, Early period** (7.441 ± 4.937)	**Contact, Early period** (7.581 ± 3.852)	**Infection, Late period** (10.444 ± 4.109)	**Contact, Late period** (10.744 ± 7.165)
**MOCK** (3.285 ± 1.905)	-	** p = 0.0099	** p = 0.0013	*** p < 0.001	** p = 0.0016
**Infection, Early period** (7.441 ± 4.937)	** p = 0.0099	-	p = 0.9254	p = 0.05545	p = 0.1166
**Contact, Early period** (7.581 ± 3.852)	** p = 0.0013	p = 0.9254	-	* p = 0.0382	p = 0.1082
**Infection, Late period** (10.444 ± 4.109)	*** p < 0.001	p = 0.05545	* p = 0.0382	-	p = 0.8787
**Contact, Late period** (10.744 ± 7.165)	** p = 0.0016	p = 0.1166	p = 0.1082	p = 0.8787	-

*p < 0.05, **p < 0.01, and ***p < 0.001

Appendix Table 3. Comparison of lung parenchymal area ratios between five study groups: results of t-tests

**Group** (Mean ± SD)	**MOCK** (18.467 ± 9.573)	**Infection, Early period** (35.133 ± 10.333)	**Contact, Early period** (54.540 ± 13.834)	**Infection, Late period** (45.102 ± 8.331)	**Contact, Late period** (34.673 ± 18.217)
**MOCK** (18.467 ± 9.573)	-	*** p < 0.001	*** p < 0.001	*** p < 0.001	** p = 0.0087
**Infection, Early period** (35.133 ± 10.333)	*** p < 0.001	-	*** p < 0.001	** p = 0.0031	0.9264
**Contact, Early period** (54.540 ± 13.834)	*** p < 0.001	*** p < 0.001	-	* p = 0.0183	*** p < 0.001
**Infection, Late period** (45.102 ± 8.331)	*** p < 0.001	** p = 0.0031	* p = 0.0183	-	* p = 0.0340
**Contact, Late period** (34.673 ± 18.217)	** p = 0.0087	0.9264	*** p < 0.001	* p = 0.0340	-

*p < 0.05, **p < 0.01, and ***p < 0.001

Appendix Table 4. Reduction in SARS-CoV-2-specific plaque formation rates via neutralized antibodies derived from the infected and contact groups of dogs

		**Days post infection (dpi)**				
**Group**	**Dog number**	**7 dpi**	**14 dpi**	**21 dpi**	**28 dpi**	**35 dpi**
Infection	Infected dog 1	69.47 ± 0.05%	93.75 ± 0.02%	96.77 ± 0.03%	92.47 ± 0.04%	96.67 ± 0.03%
	Infected dog 2	69.47 ± 0.06%	89.06 ± 0.08%	92.47 ± 0.05%	93.55 ± 0.03%	94.44 ± 0.04%
	Infected dog 3	32.63 ± 0.06%	-	-	-	-
	Infected dog 4	64.21 ± 0.08%	93.75 ± 0.06%	-	-	-
	Infected dog 5	91.58 ± 0.04%	93.75 ± 0.02%	95.70 ± 0.04%	96.77 ± 0.03%	96.67 ± 0.03%
	Infected dog 6	84.21 ± 0.03%	-	-	-	-
Contact	Contacted dog 1	3.16 ± 0.06%	85.94 ± 0.04%	88.17 ± 0.04%	95.70 ± 0.04%	92.22 ± 0.07%
	Contacted dog 2	4.21 ± 0.05%	79.69 ± 0.06%	91.40 ± 0.02%	98.92 ± 0.02%	93.33 ± 0.07%
	Contacted dog 3	6.32 ± 0.04%	-	-	-	-
	Contacted dog 4	5.26 ± 0.03%	3.13 ± 0.08%	-	-	-
	Contacted dog 5	28.42 ± 0.05%	76.56 ± 0.04%	89.25 ± 0.05%	97.85 ± 0.02%	85.56 ± 0.04%
	Contacted dog 6	32.63 ± 0.05%	-	-	-	-

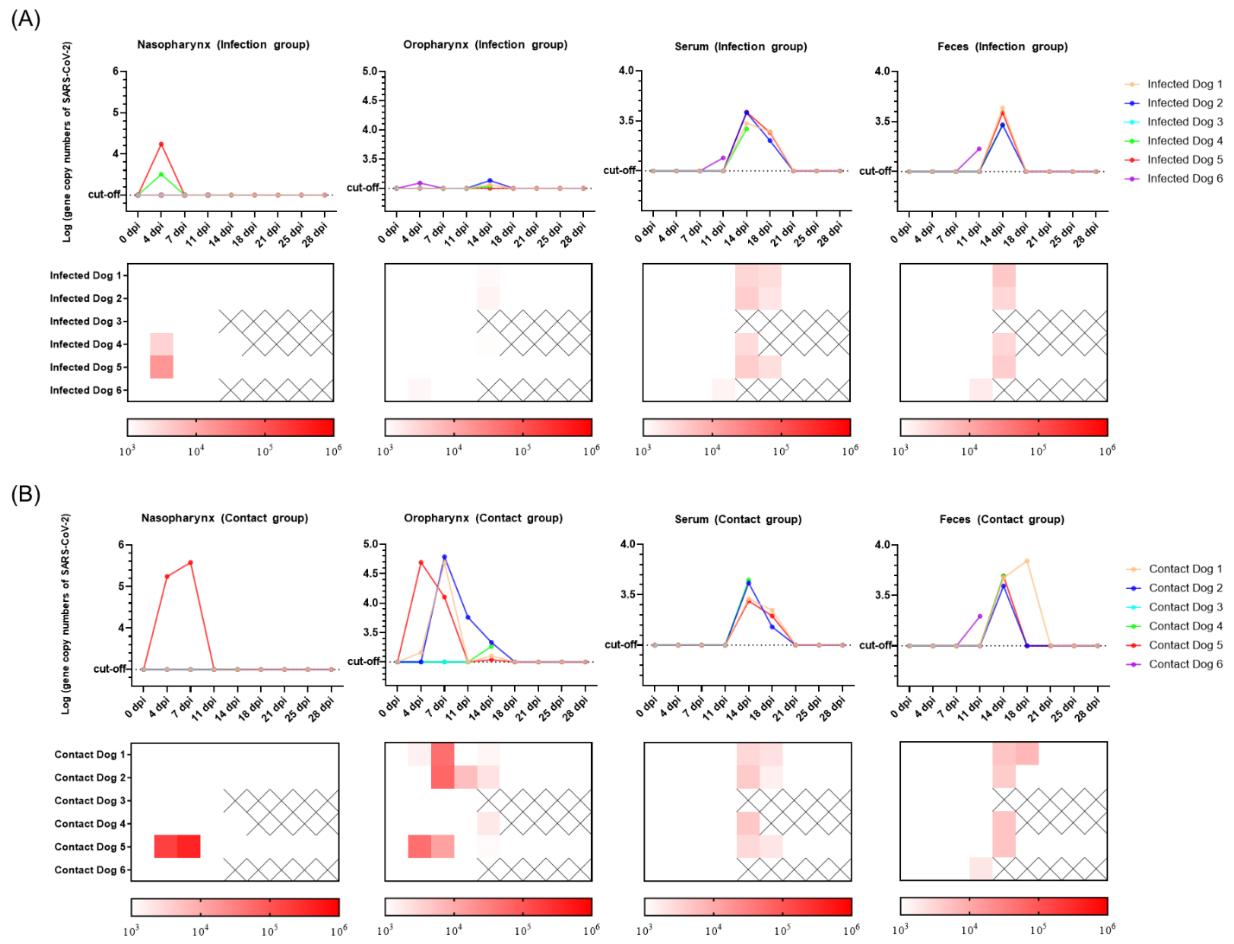

Appendix Figure 1. Detection of SARS-CoV-2 in the nasopharyngeal, oropharyngeal, and fecal swabs, and serum of infected and contact dogs for 28 days post-infection. Serum, fecal, nasopharyngeal, and oropharyngeal swabs were collected periodically from all dogs following virus inoculation. After the inactivation process (55°C, 30 min), the samples underwent further analysis for viral RNA using RT-qPCR. The Ct value corresponding to the lowest detectable copy number on the standard curve, where the fluorescence signal exceeds the background noise, was set as the cut-off value. Additionally, heat maps are presented below the line graphs to facilitate better comprehension of the same results.

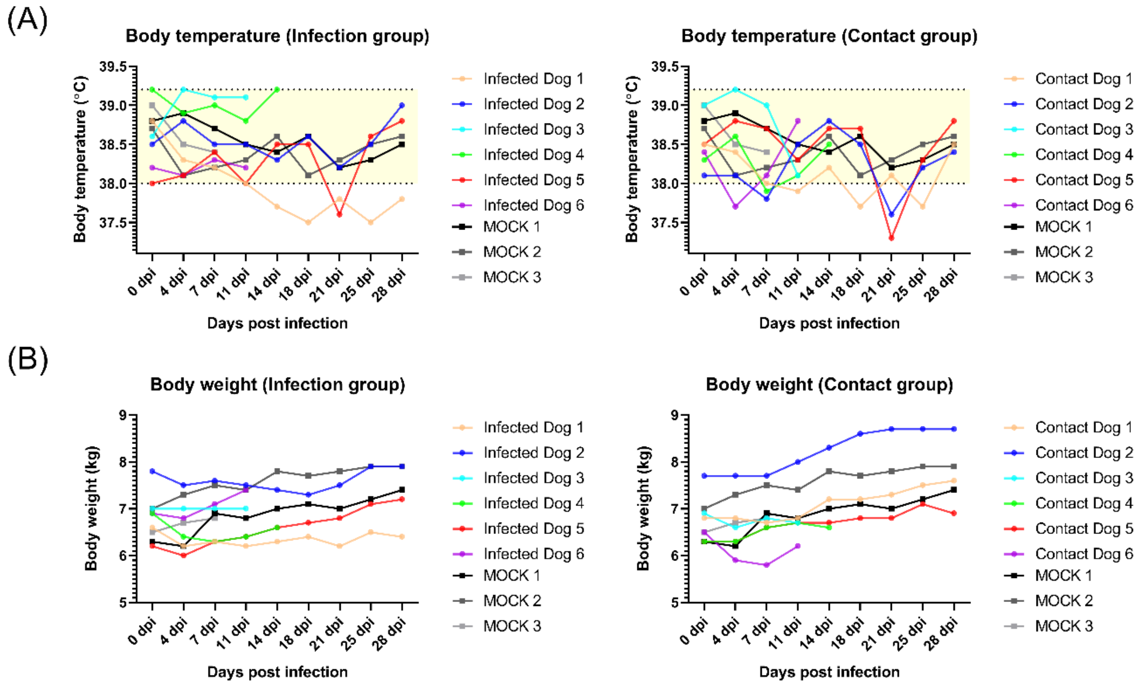

Appendix Figure 2. Changes in body temperature and weight of SARS-CoV-2-infected and contact dogs. (A) Body temperature was measured via rectal thermometry. No dogs with high fever were observed during the experiment; however, some dogs exhibited mild hypothermia. The normal body temperature range for dogs was established as 38.0–39.2°C and highlighted in a yellow box. (B) No significant changes in weight were observed after SARS-CoV-2 infection.

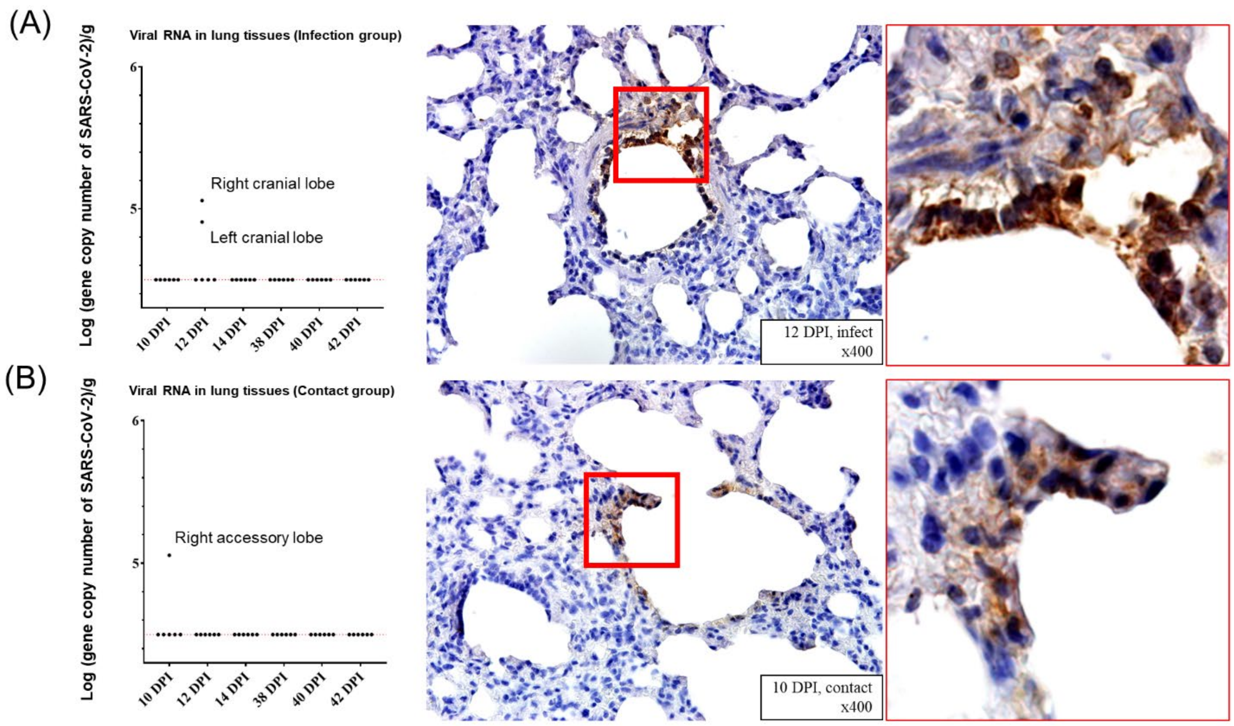

Appendix Figure 3. Detection of SARS-CoV-2 RNA in the lung tissues of infected and contact dogs and their immunohistochemistry images. Following autopsy, RT-qPCR was conducted to determine the presence of SARS-CoV-2 in the lung tissues. Among the infection group, two out of 18 early-period lung tissues tested PCR-positive for the virus (A), while one out of 18 early-period lung tissues tested PCR-positive for the virus in the contact group (B). No viral RNA was detected in the late-period tissues. IHC analysis of the PCR-positive tissues confirmed the presence of PCR-positive viral proteins.

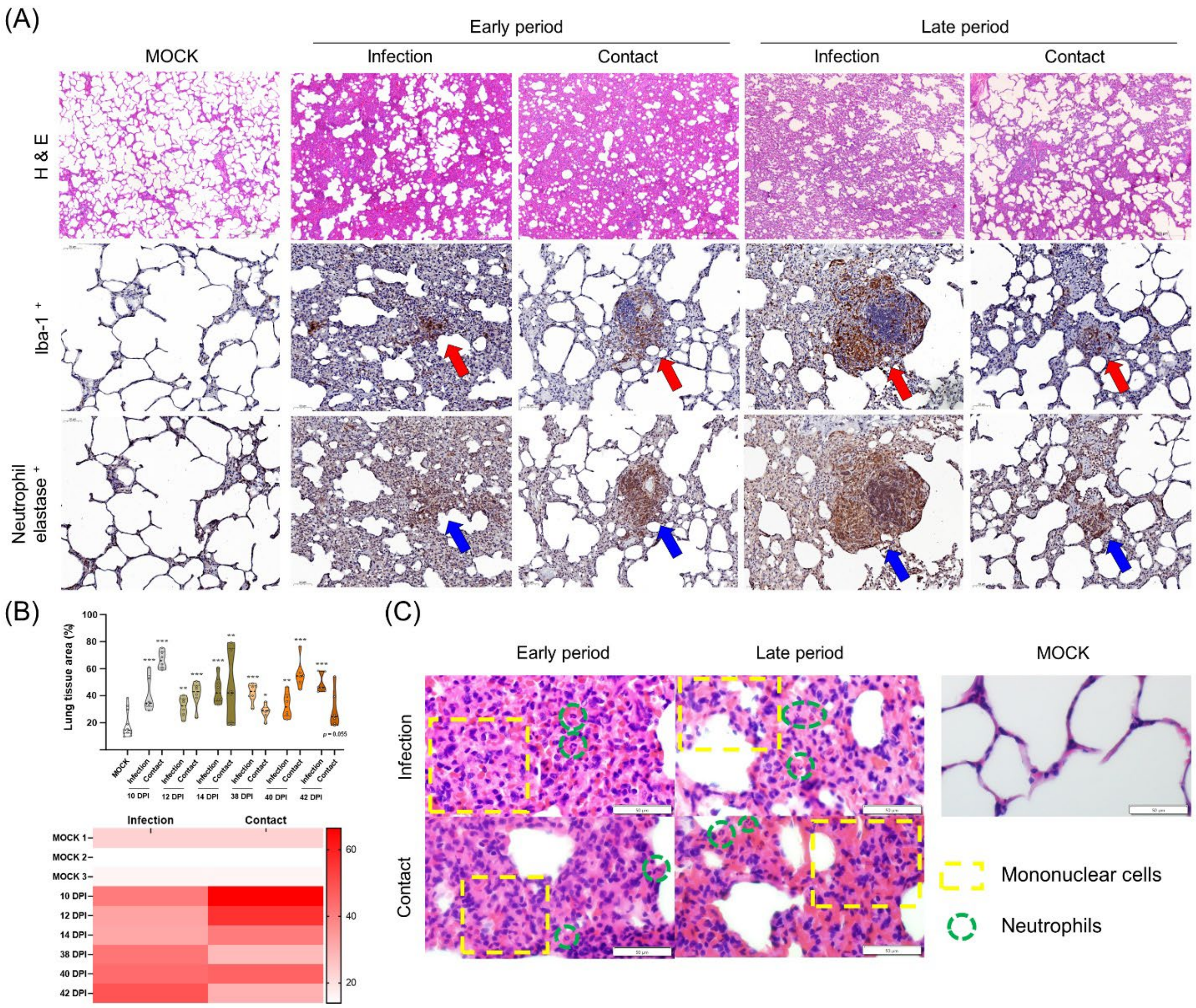

Appendix Figure 4. Thickening of the alveolar septa with interstitial pneumonia and infiltration of immune cells. (A) Histopathological changes in the lung tissue of dogs were confirmed through H&E and immunostaining. The H&E staining revealed a thickening pattern of the alveolar septum, while immunostaining showed infiltration of macrophages (red arrow) and neutrophils (blue arrow). (B) Area ratio of the lungs was calculated using the ImageJ program. Data are presented as mean ± SD with a violin plot. *$p < 0.05$, ** $p < 0.01$, and *** $p < 0.001$. (C) The image depicts a close-up view of an alveolar septum that exhibits infiltration of neutrophils (dotted green circle) and mononuclear cells (dotted yellow square).

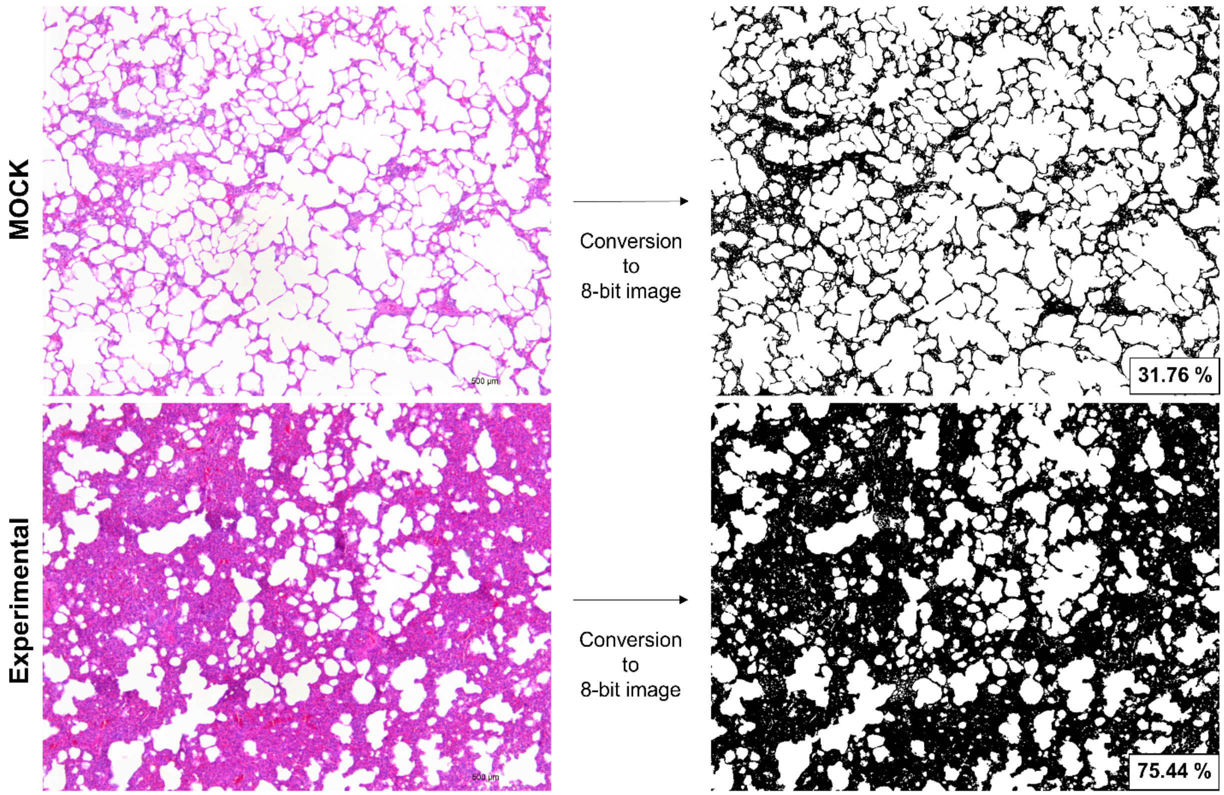

Appendix Figure 5. Quantification process of lung tissues by converting to 8-bit images. We randomly selected six parts of the H&E-stained lung tissue slides and compared the ratios with the lung tissue areas. The tissue parts of the lungs were quantified after converting to an 8-bit image. Black parts were digitized using the ImageJ program.

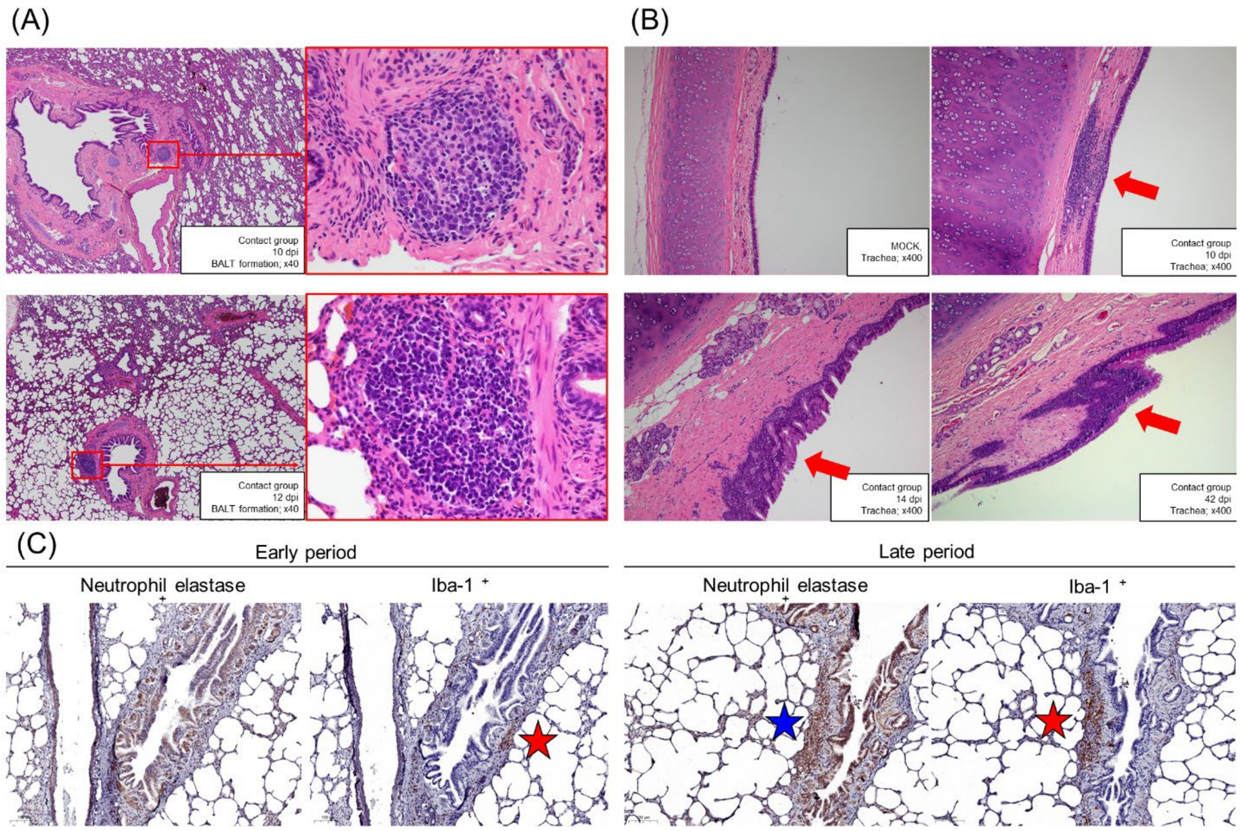

Appendix Figure 6. Histopathological changes in the lung tissues of contact dogs. (A) Bronchus-associated lymphoid tissue (BALT) formation was observed during the early infection period in contact dogs. (B) Inflammatory cell infiltration (red arrow) was also observed in the organs of dogs in the contact group during the late stages of infection. (C) In addition to BALT formation, inflammatory cell infiltration was confirmed in the bronchioles. Immunostaining revealed infiltration of neutrophil elastase-positive cells, a marker of neutrophils, during the late period (blue star). Additionally, infiltration of Iba-1-positive cells, a marker of macrophages, was observed during both the early and late infection periods (red star).

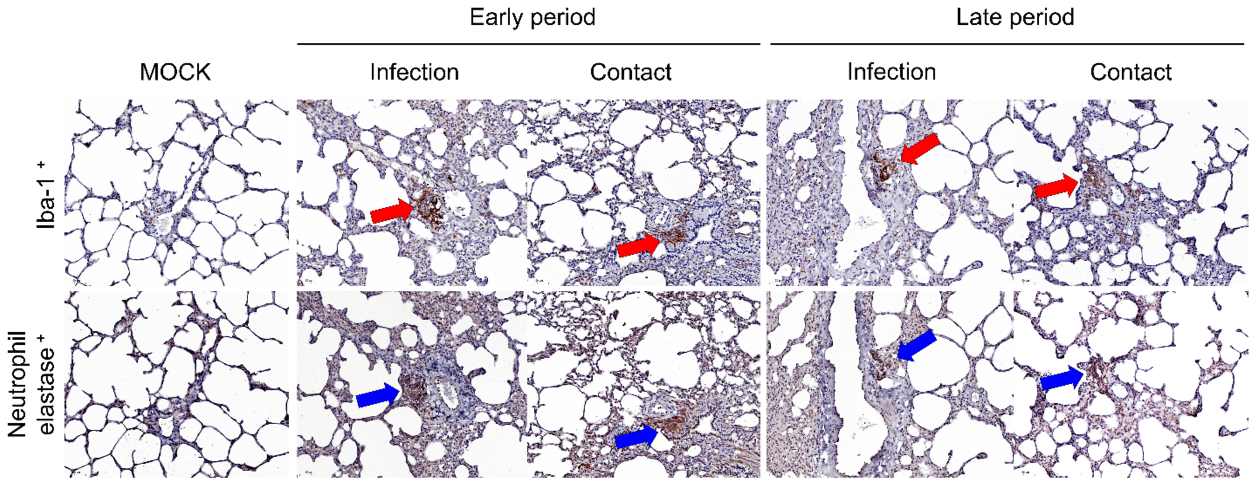

Appendix Figure 7. Infiltration of immune cells to adjacent pulmonary blood vessels. Immunostaining revealed infiltration of Iba-1-positive cells (red arrow), a marker of macrophages, and neutrophil elastase-positive cells (blue arrow), a marker of neutrophils, around the pulmonary blood vessels during both the early and late infection periods.

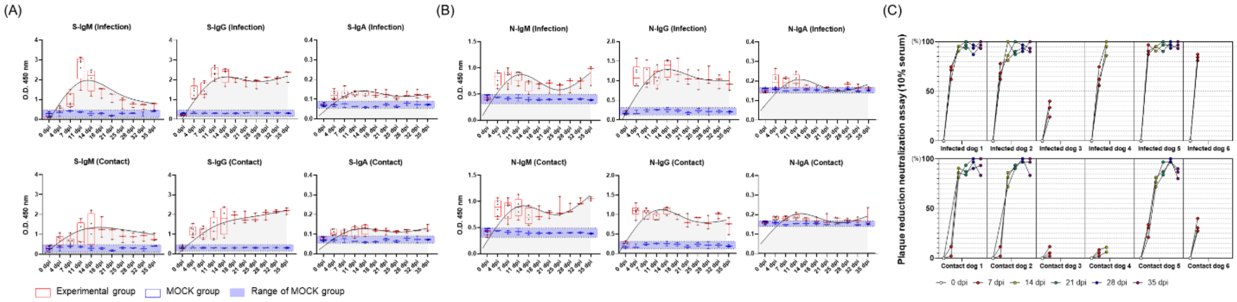

Appendix Figure 8. Seroconversion of IgA, IgM, and IgG antibodies to the spike S and nucleocapsid proteins of SARS-CoV-2. After SARS-CoV-2 inoculation, dog blood was collected at regular intervals. The response of IgA, IgM, and IgG antibodies to S protein (A) or N protein (B) in the serum is shown in the box and whisker plots (red box: experimental groups; blue box: MOCK groups). All antibody values detected in uninfected dogs are distributed within the range of the purple box. Smoothing spline curves with four knots were created in 48 segments (black). (C) The formation of neutralizing antibodies in the experimental group of dogs was evaluated using diluted serum (10-fold dilution).

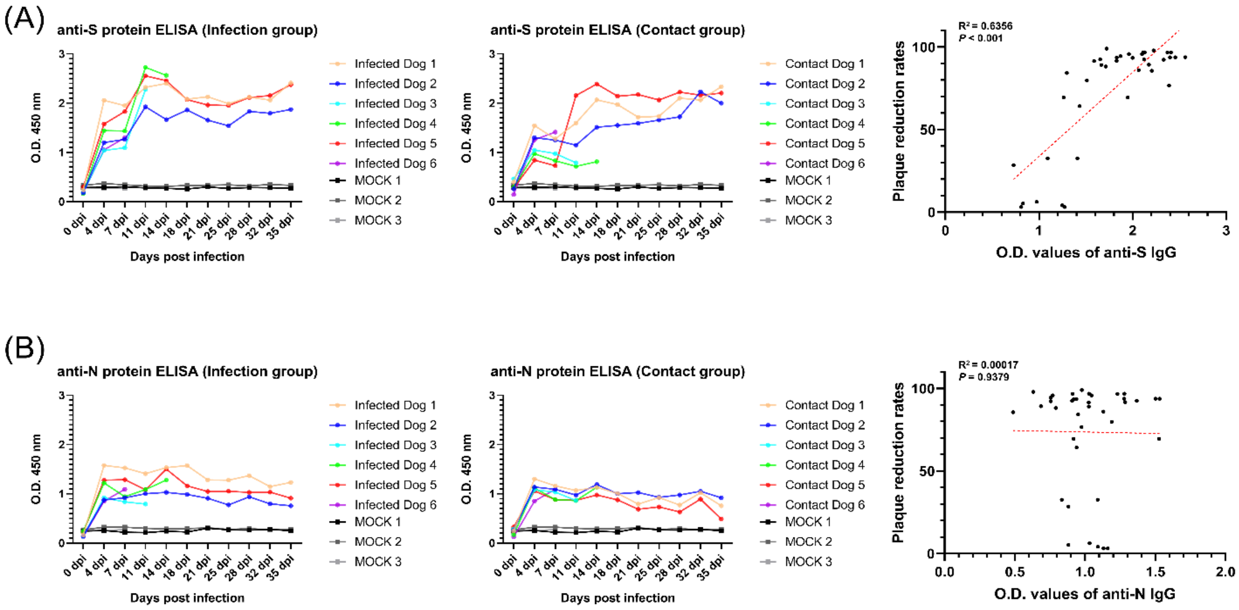

Appendix Figure 9. IgG antibody titers of individual dogs after SARS-CoV-2 infection. To determine the relationship between the formation of neutralizing antibodies and the levels of IgG antibodies for each dog (data in Appendix Figure 8), an individual IgG antibody graph and correlation plot between plaque reduction rate and O.D. values of the IgG antibody were generated.

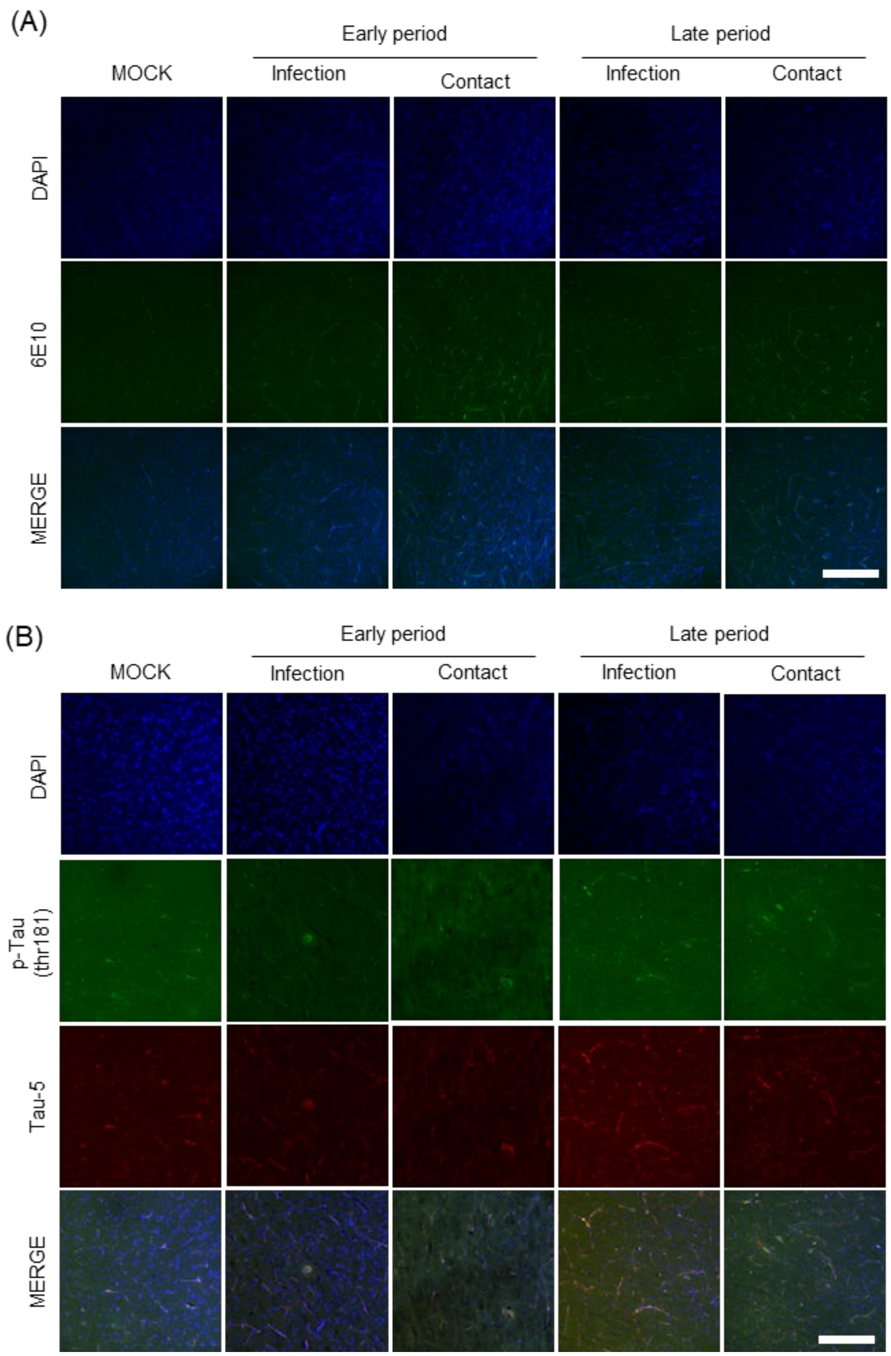

Appendix Figure 10. Amyloid β accumulation and Thr181-phosphorylated Tau were not detected in SARS-CoV-2 infected dogs. (A) Representative confocal microscopic images of 6E10 (a marker of amyloid beta, green) staining of canine brain gray matter sections derived from SARS-CoV-2-infected and contact groups at early and late infection periods. (B) Representative confocal microscopic images of phosphorylated Tau (thr181, green) and Tau-5 (total Tau, red) staining of canine brain white matter sections derived from SARS-CoV-2-infected and contact groups at early and late infection periods. The scale bar represents 200 μm.
